# Enhancing LGBTQ+ Inclusivity in an AI-Powered Sexual Health Chatbot: User-Centered Design Approach Through a Nonprofit and Academic Partnership

**DOI:** 10.2196/78621

**Published:** 2026-02-19

**Authors:** William Wibowo Liem, Elizabeth Casline, Julianna Lorenzo, Jacob D Gordon, Andrés Alvarado Avila, Attia Taylor, Nicole Levitz, Michael C O'Keefe, Karen Shum, Kathryn Macapagal

**Affiliations:** 1 Impact Institute, Northwestern University, Chicago, IL, United States; 2 College of Nursing, University of Cincinnati, Cincinnati, OH, United States; 3 Planned Parenthood Federation of America, New York, NY, United States; 4 Department of Medical Social Sciences, Impact Institute, Northwestern University, 625 North Michigan Avenue, Floor 14, Chicago, IL, United States, 1 312 503 3605

**Keywords:** sexual health, sex education, adolescents, sexual and gender minorities, artificial intelligence, stakeholder participation, digital health, AI, LGBTQ+, inclusivity, lesbian, gay, bisexual, transgender, and queer

## Abstract

**Background:**

Despite the growing use of digital platforms for sexual health education, many tools fail to meet the needs of LGBTQ+ (lesbian, gay, bisexual, transgender, and queer) teenagers, who often lack access to inclusive, affirming resources. Artificial intelligence (AI)–enabled chatbots have emerged as promising tools to address these gaps, but concerns remain around bias, usability, and trustworthiness—particularly for queer and transgender teenagers. Participatory design approaches centered around marginalized teenagers are critical to ensuring these tools are relevant, trustworthy, and equitable; yet, few studies have systematically engaged LGBTQ+ teenagers in the co-design of AI-powered sexual health interventions.

**Objective:**

This paper examines LGBTQ+ teenagers’ perceptions of Roo, Planned Parenthood Federation of America’s (PPFA) AI-powered sexual education chatbot to identify opportunities and challenges in delivering LGBTQ+-inclusive, affirming sexual health information.

**Methods:**

Embedded within Sharing Health Education Resources, a hybrid effectiveness implementation trial of a digital HIV prevention intervention for LGBTQ+ teenagers, we collaborated with PPFA to create a customized instance of Roo for integration into this study. We engaged a Youth Advisory Council comprising 15 LGBTQ+ teenagers to independently explore and interact with Roo, then gathered feedback through a week-long asynchronous discussion on a private Discord (Discord Inc) server. The research team posed open-ended questions prompting participants to reflect on Roo’s inclusivity, usability, and content priorities. We used rapid qualitative analysis organized around our research questions.

**Results:**

Participants expressed both skepticism and curiosity about AI’s role in delivering sexual health information, offering critical insights on the chatbot’s language, trustworthiness, and relevance. Teenagers identified key limitations in Roo’s inclusivity, tone, and interface, particularly around transgender-specific content, conversational depth, and stigma reduction. These findings informed targeted content updates, interface refinements, and transparency improvements, implemented by PPFA to enhance Roo for broader use. Specific changes included expanding LGBTQ+ affirming content, revising language to eliminate gendered assumptions, incorporating concrete statistics and contextualized examples to reduce stigma, and adding clearer disclosures around Roo’s AI capabilities and limitations.

**Conclusions:**

Academic and nonprofit collaborations can leverage participatory methods to enhance digital health tools in real-world contexts. LGBTQ+ teenagers served not only as testers but as co-designers, shaping the chatbot’s evolution and surfacing broader lessons about trust, AI literacy, and health equity. This study demonstrates that marginalized teenagers possess the critical insights needed to meaningfully shape AI-enabled health interventions when provided with structured opportunities for engagement. This partnership offers a scalable model for integrating community voice into the development, evaluation, and implementation of inclusive, AI-enabled health technologies.

## Introduction

LGBTQ+ (lesbian, gay, bisexual, transgender, and queer) teenagers experience significant sexual and reproductive health disparities compared to their cisgender, heterosexual peers. They face higher rates of sexually transmitted infections (STIs) and HIV, with young men who have sex with men accounting for 81% of new HIV diagnoses among teenagers aged 13-19 years in the United States [1]. Sexual minority individuals who can become pregnant are also at increased risk for unintended pregnancies due to higher rates of sexual victimization and coerced sex [2]. Additionally, LGBTQ+ teenagers are more likely to experience intimate partner violence than their heterosexual peers, with transgender and nonbinary teenagers reporting particularly high rates of victimization [3]. Without access to inclusive, affirming sexual health resources designed for their needs, these disparities risk worsening. Accordingly, inclusivity, usability, and perceived helpfulness emerge as key criteria for evaluating digital sexual health tools—determining whether LGBTQ+ teenagers feel represented, can navigate the platform, and find information meaningful for decision-making.

Access to comprehensive, inclusive sexual health education plays a critical role in equipping teenagers with the knowledge and skills to navigate consent, boundaries, and healthy relationships. Yet, school-based sexual health education in the United States remains overwhelmingly focused on cisgender, heterosexual experiences, often omitting or stigmatizing LGBTQ+ identities [4,5]. As a result, many LGBTQ+ teenagers turn to the internet to fill these educational gaps [6], seeking inclusive and affirming resources that traditional channels fail to provide [7-9]. In the United States, digital interventions have emerged as powerful tools for delivering inclusive sexual health education to teenagers. Platforms ranging from mobile apps to interactive websites offer accessible, affirming alternatives [10]. For LGBTQ+ teenagers in particular, online platforms can serve as safer spaces to access accurate information, connect with peers navigating similar challenges, and build critical knowledge around consent, relationships, and HIV prevention [11]. Evidence from interventions such as TechStep (designed for transgender teenagers) and MyPEEPS Mobile (tailored to same-sex attracted teenage boys) demonstrates how well-designed, targeted content can successfully reduce condomless sex practices [12,13]. The success of these programs underscores the importance of thoughtful, community-informed design; features such as user-friendly interfaces, engaging and culturally relevant messaging, and interactive components (eg, reminder texts or skill-building exercises) can significantly enhance user engagement and educational outcomes [14]. As digital tools continue to evolve, these insights offer a critical foundation for the next generation of technology-enabled interventions, including those powered by artificial intelligence (AI).

Incorporating AI in personalized health care is emerging as a promising avenue to further enhance accessibility and engagement with users [15]. However, the adoption of AI-driven tools in sexual health education has sparked both optimism and skepticism, particularly among LGBTQ+ teenagers. While chatbots can effectively deliver health interventions when designed appropriately with LGBTQ+ individuals, significant concerns remain about information quality, reading level appropriateness, and inconsistent accuracy [16,17]. These concerns are particularly acute for Black LGBTQ+ youth facing health care access barriers. A scoping review of generative conversational AI apps for the LGBTQ community found that while initial deployments were feasible and well-received, substantial improvements are needed in content quality and engagement [18]. These findings underscore both AI’s potential to address health disparities and the critical need for further development, formal evaluation, and community-informed design.

The “myth of AI,” as described by McAra-Hunter [19], creates unrealistic expectations that AI can universally solve complex problems, often ignoring the nuanced needs of marginalized groups. This disconnect poses risks, as AI tools are frequently developed without sufficient input from LGBTQ+ communities, leading to unrealistic expectations and potentially harmful outcomes. For example, ChatGPT (OpenAI) has been shown to use outdated or exclusionary language—assuming people with vaginas are women, and those with penises are men—and to lack sociopolitical sensitivity in its responses [20]. These findings echo broader concerns raised by Tomasev et al [21], who caution that AI systems trained on large-scale datasets without community-informed oversight risk encoding and amplifying societal biases, particularly those affecting gender and sexual minorities. Moreover, a study by Hopelab found that LGBTQ+ teenagers are more likely than their cisgender and heterosexual peers to avoid AI tools due to concerns about inaccuracies and biases [22]. Integrating teenagers’ input into the design process can enhance trust, usability, and the overall experience of AI-powered interventions, ensuring they align more closely with the needs of the communities they aim to serve. However, queer youth are rarely positioned as designers of AI health tools, despite their unique expertise in navigating heteronormative and cisnormative health care systems [23].

Despite the promise of digital and AI-powered tools, research-driven innovations often face a persistent research-to-adoption gap, where effective interventions fail to achieve sustained public health impact. It is estimated that it can take an average of 17 years for research findings to be translated into real-world practice, with only a small fraction ever implemented at scale [24]. To address this gap, it is critical to embed considerations of implementation and sustainability early in the design process, especially for teenager-centered tools. This is especially important for LGBTQ+ teenagers who often rely on digital tools to access affirming information and support that may be unavailable in their offline environments.

Participatory research approaches are particularly well-suited for engaging marginalized populations such as LGBTQ+ teenagers, as they center community expertise, address power imbalances inherent in research, and ensure interventions reflect the lived realities of those most affected [25]. By involving teenagers as co-designers rather than passive recipients, participatory methods not only improve intervention relevance and acceptability but also build trust with communities that have historically experienced harm or exclusion from medical institutions [26]. A key implementation is developing nonprofit and academic partnerships that bridge the research-to-adoption gap while facilitating rigorous co-design and evaluation methods. Evidence shows that involving teenagers in intervention planning leads to better reflection of their needs and perspectives while addressing power imbalances in decision-making [27]. Coproducing interventions with teenagers increases their sense of ownership and supports adoption, with 1 review reporting more than a 2-fold increase in self-reported use of sexual and reproductive health services [28]. For these partnerships to maximize lasting public health benefit, there is a high need to prioritize cocreation, effectively manage expectations early, and integrate implementation planning from the outset.

**Figure 1.**
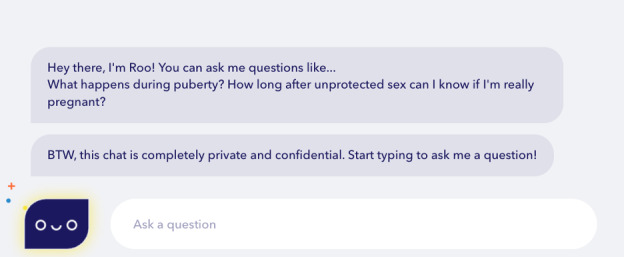
Roo chatbot interface (Planned Parenthood, accessed May 31, 2025). Roo, Planned Parenthood Federation of America’s free, web-based AI-powered sexual education chatbot. The interface features example prompts (eg, “What happens during puberty?” and “How long after unprotected sex can I know if I’m really pregnant?”), a privacy assurance message, and an open text input field for user-generated questions. Roo’s database covers topics including abortion, birth control, relationships, LGBTQ+ health, and symptoms. AI: artificial intelligence; BTW: by the way; LGBTQ+: lesbian, gay, bisexual, transgender, and queer.

To illustrate how aligning missions across sectors can accelerate impact, we describe the nonprofit and academic collaboration between Planned Parenthood Federation of America (PPFA) and Northwestern University aimed to enhance Roo (Figure 1), an existing, publicly available PPFA-operated AI-powered platform, through a week-long asynchronous discussion with LGBTQ+ teenagers on Discord.

## Methods

### Study Design

This study was conducted as part of a hybrid type 1 effectiveness implementation randomized controlled trial examining the effectiveness of a digital text-based HIV prevention intervention called Sharing Health Education Resources (SHER) compared to an information-only control among LGBTQ+ teenagers. SHER builds on an earlier text-messaging intervention called Guy2Guy (G2G), a text messaging-based HIV prevention program to deliver educational and skill-oriented information to male teenagers who identify as gay, bisexual, and/or queer [29]. Within SHER, Roo, Planned Parenthood’s AI-powered sexual education chatbot, was adapted and evaluated as a component intervention. For the purposes of this paper, we focus specifically on Roo and report findings from Youth Advisory Council (YAC) feedback about its inclusivity, usability, and trustworthiness.

**Figure 2.**
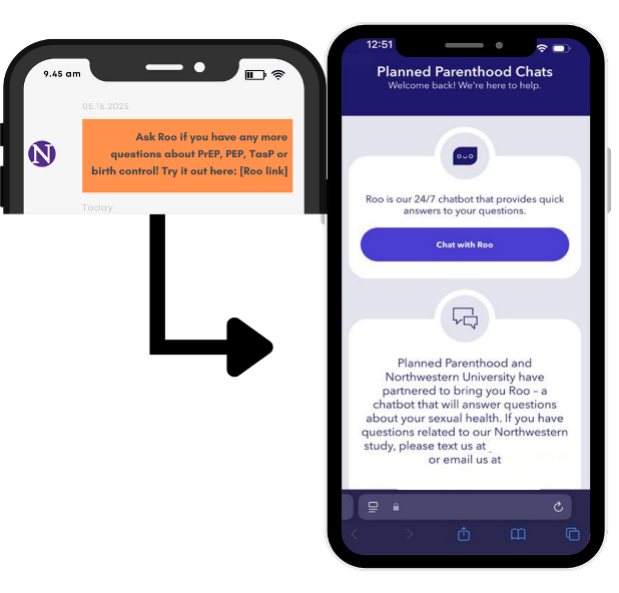
Integration of the Roo chatbot into the SHER digital HIV prevention intervention for LGBTQ+ teenagers. The screenshot demonstrates how participants who enrolled in the SHER text messaging intervention (left panel) received a prompt directing them to Roo, Planned Parenthood’s AI-powered sexual education chatbot (right panel). The customized Northwestern University instance of Roo was accessible via a dedicated microsite and provided on-demand sexual health information, complementing SHER’s structured intervention content. AI: artificial intelligence; LGBTQ+: lesbian, gay, bisexual, transgender, and queer; PEP: post-exposure prophylaxis; PrEP: pre-exposure prophylaxis; SHER: Sharing Health Education Resources; TasP: Treatment as Prevention.

The original G2G intervention featured an on-demand texting function, G2Genie, that allowed participants to request additional information by sending keywords (eg, texting “G2Genie relationships” would prompt a curated set of messages about relationship skills). Building on this concept—and recognizing the growing emphasis on personalization and rapid delivery in digital health interventions—SHER planned to adapt this feature by developing a bespoke sexual education chatbot feature tailored to the specific needs of LGBTQ+ teenagers [30,31]. The goal of the chatbot was to enable participants to ask follow-up questions and receive individually tailored responses across a broader range of sexual health topics than those included in the SHER text messages (Figure 2). By collaborating with a nonprofit resource already integrated into public health infrastructure, Northwestern University was able to accelerate translation, broaden reach among diverse teenage audiences, and leverage existing dissemination pathways to enhance both impact and equity. Rather than building a proprietary chatbot accessible only to study participants, our team strategically partnered with an existing, publicly available platform, PPFA’s Roo.

Roo is an AI-powered chatbot designed to provide confidential, accessible, and medically accurate sexual health information. Roo’s responses are developed by Planned Parenthood’s content and education teams to maintain a friendly, nonjudgmental tone through intentional strategies such as using conversational language (eg, “You deserve fun, comfortable, and pleasurable sex”), avoiding clinical jargon, affirming diverse experiences as normal, and framing sensitive topics without moral judgment. For instance, rather than emphasizing risk, Roo normalizes STI testing as routine care (eg, “Getting tested is often as easy as peeing in a cup at a health center”) and highlights that “most of the time, STDs don’t show symptoms, so the only way to know for sure is to get tested.” Accessible via the PPFA website [32], Roo allows users to ask free-text questions and responds immediately with a nonjudgmental answer and a list of additional resources for the user to engage with.

Roo’s development is grounded in the Unified Theory of Behavior, which integrates multiple behavioral theories to explain and influence health behaviors through attitudes, normative beliefs, self-efficacy, and behavioral intention [33]. Roo’s content and education teams crafted responses to both provide information while supporting users in setting behavioral intentions. This application of the theory is operationalized through several strategies: presenting pros and cons to support informed decision-making, acknowledging emotions users may experience, connecting users to health care resources, and providing referrals to partner organizations. For example, when discussing contraception options, Roo provides balanced information about effectiveness and side effects while prompting users to consider their personal priorities and next steps, such as scheduling a clinic appointment. All educational content on Planned Parenthood’s website, including Roo, reflects this framework by helping users identify action plans and set intentions for next steps in their sexual health journey. To our knowledge, Roo is the only sexual and reproductive health chatbot that explicitly applies the Unified Theory of Behavior in its design and content strategy, distinguishing it from other AI-powered tools focused primarily on information delivery rather than behavioral support.

Roo uses natural language processing to scan each question for keywords to trigger the correct answer or send the user to another resource that can answer their question. Natural language processing uses machine learning and deep learning models to recognize patterns in the human language. Roo is a Planned Parenthood proprietary product that draws exclusively from a curated library of responses designed specifically for Roo users. All content is crafted and consistently iterated upon by Planned Parenthood’s technology and sex education leadership teams, with all responses conforming to the federation’s medical standards and guidelines. This human-curated approach ensures that responses maintain medical accuracy, age-appropriateness, and alignment with PPFA’s mission of providing inclusive, affirming sexual health education. Topics in Roo cover a wide range of sexual and reproductive health topics. The questions that Roo can answer are limited by the database of content that Roo is able to pull from. The content team at Planned Parenthood uses data from Roo, other digital products, and user experience research to add common and frequently asked questions and their responses into Roo. If Roo cannot answer a user’s question, it will let them know and offer alternatives, including PPFA’s anonymous live chat with real health educators, as well as links to PPFA’s blog and other educational resources.

In 2023, Roo facilitated approximately 640,000 sessions [34]. Roo offers a complementary approach to SHER’s content and structure. Whereas SHER delivers structured, sequential educational messages with preplanned daily content, Roo is designed to offer on-demand, conversational access to medically accurate, affirming, and developmentally appropriate sexual health education. Roo’s database covers a broad range of topics relevant to teenagers, including safe sex practices, consent, healthy relationships, LGBTQ+ health, and navigating health care systems.

This complementary functionality made Roo a promising addition to SHER, enhancing the program’s ability to support LGBTQ+ teenagers’ diverse information needs without duplicating content delivery. For PPFA, Roo has provided an opportunity to align its mission with innovative delivery models; however, implementing an AI-enabled tool that adequately meets the needs of LGBTQ+ teenagers has required technical and evaluation infrastructure beyond PPFA’s in-house capacity. By collaborating with an academic institution, PPFA was able to benefit from Northwestern University’s methodological expertise to co-design an intervention evaluation to receive actionable recommendations for iteratively refining Roo’s responsiveness to LGBTQ+ teenagers’ needs.

### Academic and Nonprofit Partnership Collaborative Development Process

We sought to develop an academic and nonprofit partnership that advances both scientific rigor and real-world impact in the development of inclusive digital health innovations. Below, we describe the development of this academic and industry partnership using Proctor et al [35] domains for systematically specifying implementation strategies.

Speaking of actors, the partnership was initiated by the Northwestern University research team, who became aware of PPFA’s Roo chatbot and were connected through a mutual colleague. Key actors included academic investigators, digital health researchers, product managers, and health education specialists from both institutions.

For action*,* we developed a collaborative relationship with PPFA that required iterative conversations to align organizational goals, resources, and capacities. Academic and industry collaborations in digital health often face challenges around aligning timelines, balancing rigor and innovation, and navigating competing priorities [30]. Early meetings focused on relationship-building, clarifying organizational priorities, and identifying areas of mutual benefit. Specific actions included assessing the fit between Roo and the SHER intervention, mapping resource needs, identifying technical constraints, establishing intervention goals across both teams, and drafting and negotiating formal agreements, including a nondisclosure agreement and data use agreement to facilitate ethical collaboration and data sharing. Given the reciprocal benefits identified, both parties agreed that there would be no financial exchange.

Subsequent meetings explored integration pathways, ultimately deciding to create a standalone customized microsite version of Roo for SHER to facilitate tracking participant engagement and allow key modifications to maintain intervention integrity. These changes included removing the option to talk to a live health educator and directing users who needed help with Roo to call the SHER study team rather than chat with PPFA. This instance was built and maintained by PPFA’s product team, with all feedback integrated into both the SHER study instance and the publicly facing version of Roo.

Once the partnership was established, Northwestern University engaged its YAC—a diverse group of LGBTQ+ teenagers to test Roo and provide structured feedback on its content, user experience, and inclusivity.

Of temporality and dose, partnership development occurred over 6 months (February to July 2023) with monthly formal meetings supplemented by asynchronous collaboration, with higher-frequency early communication for trust-building and later focus on YAC feedback and platform customization.

Of target and justification, the overarching target was to integrate Roo into SHER in a way that enhanced accessibility, inclusivity, and personalization of sexual health resources for LGBTQ+ teenagers. PPFA aimed to expand its reach to LGBTQ+ teenagers, while Northwestern University aimed to apply participatory design principles centering LGBTQ+ youth voices. Partnering with an existing, trusted platform embedded in public health infrastructure allowed the project to accelerate translation and broaden access beyond this study’s sample.

### Ethical Considerations

Concerning ethics approval, all study procedures were approved by the Northwestern University Institutional Review Board (study number: STU00217358) with a waiver of parental consent, given the potential risks of requiring guardian permission for LGBTQ+ minors.

Of informed consent, for minors, informed assent and a capacity-to-consent assessment were required. Eligibility was confirmed through screening forms and one-on-one video calls with study staff, which also served to review participation expectations and privacy protocols.

Of privacy and confidentiality, the YAC operates on a private, invite-only Discord server, where participants can use pseudonyms to protect their identity. Study staff moderated the server to ensure a safe, respectful environment. Participants could withdraw from this study at any time without penalty, and all data were stored securely in compliance with institutional data protection standards. The principal investigator is a licensed clinical psychologist with expertise in LGBTQ+ adolescent health, ensuring clinical oversight of all activities involving sensitive sexual health discussions.

Concerning compensation, participants received US $30 in digital gift cards per month for active participation, with opportunities to earn up to US $200 annually for optional activities.

Of the use of images, no identifiable participant images are included in this manuscript or supplementary materials.

### Recruitment

As part of the SHER effectiveness implementation trial, LGBTQ+ teenagers were recruited through paid Meta (Facebook or Instagram) advertisements and an institutional participant registry. Interested teenagers were directed to a secure REDCap (Research Electronic Data Capture; Vanderbilt University) screening survey to complete eligibility questions and provide contact information. To reduce impostor enrollment, identity verification included a brief video call assessing personal information and comprehension of study details [36-38]. Following a virtual orientation session conducted via Zoom (Zoom Communications, Inc), eligible participants were invited to a private Discord server, an instant messaging platform that supports multimedia messaging services, voice, video, and multimedia communication.

Eligible participants were aged 13‐18 years, identified as sexual and/or gender minority, lived in the United States or US territories, and were able to read in English at an eighth-grade level. Recruitment sought to oversample transgender or gender-diverse and racial or ethnic minority teenagers to ensure diverse feedback. Recruitment occurred between January 2023 and July 2023 and was conducted entirely online with no in-person involvement. The YAC ultimately included 15 LGBTQ+ teenagers aged 15‐18 (mean 16.9) years (see Table 1 for demographics). For additional details on the YAC methods, see the study by Gordon et al [39].

**Table 1.** Demographic characteristics of the YAC^a^ participants, including age, race or ethnicity, gender identity, and sexual orientation. The YAC comprised 15 LGBTQ+^b^ adolescents (aged 15-18, mean 16.9 years) recruited from across the United States between January and July 2023 via paid social media advertisements and an institutional participant registry. The sample included 9 (60%) gender-diverse teenagers and 6 (40%) teenagers of color. All participants identified as sexual minorities, with the majority identifying as bisexual, pansexual, or gay.

Category	Count (n=15)
Age (years), n (%)	
15	1 (7)
16	3 (20)
17	8 (54)
18	3 (20)
Race or ethnicity, n (%)	
White	9 (60)
American Indian or Alaska Native	2 (13)
Black or African American	1 (7)
Asian	1 (7)
Mestizo Latin American	1 (7)
Not disclosed	1 (7)
Hispanic or Latino	3 (20)
Gender identity, n (%)	
Cisgender man or boy	6 (40)
Transgender man or boy	3 (20)
Transgender woman or girl	4 (27)
Gender nonconforming	1 (7)
Questioning gender identity	1 (7)
Sexual orientation, n (%)	
Bisexual or pansexual	7 (47)
Gay	5 (35)
Queer	2 (14)
Questioning	1 (7)

a YAC: Youth Advisory Council.

b LGBTQ+: lesbian, gay, bisexual, transgender, and queer.

### Participatory Feedback Process With LGBTQ+ Teenagers

The YAC is a standing, Discord-based advisory group of LGBTQ+ teenagers who provide structured input across multiple digital health projects. Before the Roo evaluation, participants reviewed intervention materials and were advised on website features, familiarizing them with research goals and developing confidence as co-designers. The Roo evaluation was therefore embedded within a broader program of participatory engagement, with inclusivity, usability, and perceived helpfulness as central evaluation criteria.

The participatory feedback process was informed by a research-through-design orientation study, which treats interventions as iterative, generative artifacts shaped through ongoing engagement and inquiry [40]. Rather than approaching Roo as a static digital tool to be evaluated in isolation, we conceptualized it as a dynamic prototype whose development was guided by LGBTQ+ teenagers’ feedback and contextualized within their lived experiences. This orientation supported creativity by encouraging teenagers to propose new content areas (eg, gender-affirming care and sex toy safety) and interface redesigns (eg, category-based navigation and dark mode), which moved beyond minor adjustments to reimagining Roo’s functionality. It also enhanced responsiveness by enabling rapid incorporation of feedback into both the SHER-specific and public-facing versions of Roo, ensuring that participants saw their input translated into visible changes. Finally, research through design positioned social and structural factors (eg, stigma in school-based sex education, barriers to gender-affirming care, and mistrust of AI systems) as central design considerations, shaping not only what content was added but also how information was framed to reduce harm and foster trust.

To prepare for the use of Roo for the intervention, the Northwestern University team and PPFA were engaged over a period of 5 months to create a Northwestern University–specific instance of Roo that would be used in the intervention. This included establishing a data use agreement, removing the health educator feature, which could be considered contamination with RCT outcomes given its behavioral component, and adding study contact information. The Northwestern University instance of Roo was shared with the YAC.

The YAC provided feedback on all components of the SHER intervention design and implementation over approximately 18 months and was focused on providing Roo feedback over 1 week. As part of this evaluation, our analysis was guided by three research questions: (1) What do LGBTQ+ teenagers perceive as gaps and/or limitations in Roo’s current content, particularly regarding inclusivity and relevance to their lived experiences? (2) How do LGBTQ+ teenagers evaluate the usability, tone, and format of Roo’s chatbot interface? (3) Under what conditions do LGBTQ+ teenagers find chatbots such as Roo helpful or unhelpful for accessing sexual health information?

To address these questions, participants were invited to interact with Roo independently and provide feedback on its usability, inclusivity, and perceived helpfulness.

Over the course of 1 week, the research team posted 3 open-ended questions in the Discord server to guide feedback. These questions prompted participants to (1) explore Roo and share initial impressions; (2) reflect on inclusivity, especially for LGBTQ+ and transgender and gender-diverse users, and suggest improvements to Roo’s interface and structure; and (3) identify additional topics Roo should cover more effectively, such as sexual assault, miscarriage, sex toys, and gender inclusivity. Participants had 7 days to respond to any or all questions at their own pace.

Each question was posted in the main research channel and was accompanied by a dedicated discussion thread to organize all responses. Participants provided written replies, reacted to peers’ comments using emojis, and engaged in follow-up exchanges within threads. Emoji reactions were used as indicators of consensus or shared resonance across YAC members.

Data for this study consisted of text responses, emoji reactions, and threaded follow-up conversations. After data collection, raw Discord content was organized into summaries by question, capturing recurring themes and illustrative quotes. In line with member-checking practices, summaries were shared with YAC members for review before being provided to the Roo team [41].

### Data Analysis

We used rapid qualitative analysis (RQA) to conduct content analysis to synthesize participant insights across our 3 research questions. RQA is an increasingly adopted approach in applied health research contexts, emphasizing structured data summarization over line-by-line coding to generate timely, actionable findings [42]. RQA is particularly well-suited for participatory projects that seek to elevate community voices while maintaining momentum in design iteration cycles [43].

Consistent with the content analysis approach, our RQA focused on a close reading of the text to identify substantive feedback that could inform actionable design improvements rather than developing emergent themes through open coding [44]. As such, our code development process prioritized capturing concrete, implementable recommendations within a time-bounded participatory design framework, an approach suitable for applied health intervention research where timely translation is essential.

The analysis was conducted collaboratively between members of the Northwestern University research team and PPFA’s digital content strategy team to ensure findings were both academically rigorous and actionable for implementation. The analytic team included the lead author (WWL, MSW, and a doctoral student with training in qualitative methods and LGBTQ+ health), a research assistant professor with expertise in implementation science and LGBTQ+ adolescent health (EC, PhD), and a research project manager with experience in community-based participatory research (AAA, MPH).

The lead author conducted the initial analysis, organizing participant feedback into a matrix where each row represented a unique participant and each column corresponded to 1 of the 3 research questions. Feedback was organized to identify substantive codes such as specific critiques, suggestions, and design recommendations that could inform actionable improvements. Responses that addressed multiple research questions were mapped across domains to reflect the interrelated nature of the data. The lead author then conducted a systematic review of the coded content to identify recurring patterns, frequently mentioned issues, and high-priority recommendations, revisiting the data for each research question domain and identifying the most actionable codes grounded in participant language. This process emphasized iterative refinement, prioritizing content with the strongest support and clearest implementation pathways and was conducted until no new substantive actionable content codes were identified. Particular attention was given to points of consensus—where feedback was echoed or affirmed across participants—as well as divergence, where responses reflected differing or underrepresented perspectives. Agreement indicators such as “likes” and emoji reactions were systematically documented as they signaled areas of shared resonance and helped prioritize implementation efforts.

The preliminary analysis was reviewed collaboratively by the Northwestern University analytic team (WWL, EC, and AAA) to ensure accuracy, comprehensiveness, and alignment with the research questions. Following this internal review, the refined findings were shared with PPFA’s Associate Director of Digital Content Strategy (AT), who brought expertise in sexual health education, digital product development, and youth engagement. The cross-organizational team then conducted a collaborative review to (1) validate themes and ensure they accurately reflected user feedback and (2) translate findings into concrete, implementable content and design changes. PPFA’s implementation process systematically prioritized changes based on frequency of feedback, level of participant endorsement (indicated by likes or reactions), feasibility, and alignment with organizational capacity for rapid implementation. This partnership approach directly informed real-world product improvements.

## Results

### Overview

The YAC provided feedback across three domains mapping to our research questions: (1) content gaps and relevance, (2) design and usability, and (3) trust and AI transparency.

### RQ1: Content Gaps, Depth, and Relevance

#### Content Depth and Specificity

YAC participants consistently emphasized the need for Roo to go beyond foundational sexual health content and engage more meaningfully with topics specific to LGBTQ+ teenagers. While Roo provided accurate general information, participants found its responses insufficiently nuanced for addressing the kinds of “medium-depth” questions they were most likely to ask. These questions, while not highly clinical, often reflected real-life scenarios central to their identities and relationships. Several participants specifically flagged the absence of content on sexual assault, miscarriages, the safe use of sex toys, and queer-specific sexual practices. One highly endorsed comment by 5 other council members summarized this concern:

*Why doesn’t Roo have any clickable question buttons about sexual assault, sex toys, and miscarriages*.

[P1, 5 likes]

This reflects a broader expectation among teenagers that a credible sexual health tool should anticipate sensitive topics and proactively make them accessible, rather than placing the burden on teenagers to generate the correct wording. Others echoed this sentiment, highlighting that Roo did not adequately respond to moderately detailed prompts related to LGBTQ+ health, with 1 participant noting, “For a lot of medium-depth questions even related [to sexual health], it didn’t have a reply” (P4, 4 likes).

#### Gender-Affirming Care and Transgender Inclusion

A particularly salient gap concerned Roo’s limited content on transgender and nonbinary health needs. Participants described the absence of information on gender-affirming care (eg, hormone therapy, safe binding practices, and navigating health care as a transgender person) as both alienating and harmful. As P9 explained,

*It’s supposed to be for transgender people like myself… but it’s not so inclusive, which would deter me from reading further most times*.

[P9]

This sense of exclusion was compounded by the broader difficulty teenagers described in accessing accurate, affirming sexual health information elsewhere. As 1 participant put it, “It’s so hard to get accurate queer transgender sex ed” (P1, 6 likes), highlighting that Roo’s shortcomings do not exist in isolation but within an already sparse landscape of transgender-inclusive health resources.

Taken together, these responses reveal that transgender-specific content is not a secondary feature but a core component of trust-building for this population. Its absence signals that the platform may not fully see or support them. Including gender-affirming information is therefore not only about comprehensiveness, but it is also about ensuring that transgender teenagers can see their realities reflected in the resource and feel affirmed and empowered to engage with it for their sexual health needs.

#### Reducing Stigma in Health Information

Participants emphasized the importance of presenting sexual health information in ways that actively reduce stigma, particularly around STIs and developmental norms. Teenagers called for content that normalizes diverse experiences rather than pathologizing them. As one participant noted, “We need info that gets rid of stigma on STIs” (P1, 2 likes), underscoring the need for nonjudgmental, affirming messaging.

At the same time, participants highlighted how even well-intentioned information could inadvertently reinforce stigma when framed without sufficient context. P3 reflected:

*It just completely negates the ‘ oh it’s okay you can take your time’ message… it may also pressure someone to have [sex] by 17, which is awful*.

[P3, 3 likes]

Rather than reassuring teenagers that there is no single timeline for sexual experiences, content that references normative milestones without careful framing was seen as inadvertently pressuring or alienating. Participants also expressed frustration with overly vague or evasive responses to personal questions. As P4 explained, “If you’re gonna say something like ‘everybody is different,’ then I’d rather just not get an answer… I’d like to see statistics… instead of just being told it depends” (7 likes).

Such comments highlight the risk of vague responses not only being unhelpful but also eroding trust in the platform’s authority. These insights suggest that effective stigma reduction requires more than inclusive language. It demands concrete, contextualized information that normalizes a range of sexual health experiences and empowers teenagers to feel validated, rather than judged, in their individual journeys.

### RQ2: Design and Usability

#### Conversational Functionality and Interface

Participants had mixed reactions to Roo’s chat-based format. While some appreciated its friendly, conversational tone, others found it cumbersome for retrieving information efficiently. Some teenagers likened the chatbot to a “complicated FAQ” (P8, 3 likes) where the conversational structure introduced unnecessary layers of interaction to access straightforward answers. As 1 participant explained:

*It doesn’t really feel like a chatbot if you’re limited to pre-written questions*.

[P4]

To address these concerns, participants proposed alternative interface designs that would offer greater flexibility, speed, and user control. Several teenagers suggested repositioning the chat input box to the bottom of the screen, aligning with familiar web design conventions and streamlining user interactions. Others advocated for adding features such as dark mode to enhance visual accessibility, particularly for users who prefer reduced screen brightness during extended sessions. The idea of categorizing topics into a dropdown menu structure (rather than relying solely on “popular questions”) was seen as critical to improving navigation and reducing the frustration associated with multiple conversational turns.

**Figure 3.**
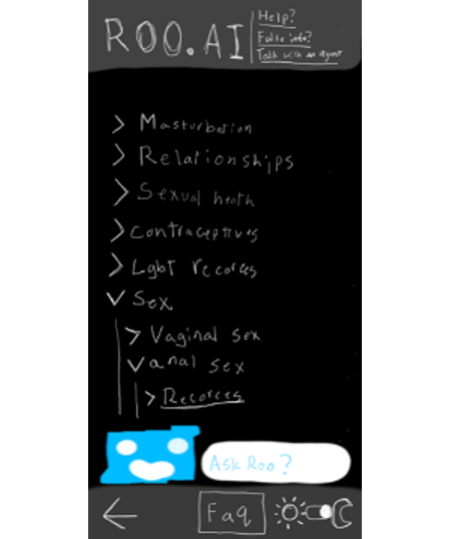
Alternative interface mockup created by P9 for Roo. The proposed redesign features a hierarchical dropdown menu structure (eg, “sex”→“vaginal sex”→“resources”) to improve navigation efficiency, dark mode for visual accessibility, a FAQ section, and an “ask Roo” chat function. This design reflects teenagers’ critiques of Roo’s original chat-based interface as cumbersome for information retrieval and their desire for categorized topic organization rather than conversational navigation. AI: artificial intelligence; FAQ: frequently asked question.

One participant (P9) proposed a comprehensive redesign that combined esthetic appeal with functionality, illustrating their vision through a self-created layout (Figure 3). Their design emphasized the importance of reducing friction in the user experience, advocating for organized topic categories (eg, “Sex>Vaginal sex>Resources”), easy-to-access FAQs, and a visually appealing, dark mode interface. Beyond esthetics, they stressed the need for Roo to maintain credibility by linking responses to trusted sources and providing external resources for users who wished to learn more. P9 explains:

*People don’t come to the AI like a social media platform—they come to ask questions about sexual health… a category dropdown that leads into a list of questions gives a more balanced system where everyone gets the same answer*.

[P9]

In addition to these interface suggestions, P9 proposed backend enhancements to strengthen Roo’s reliability and responsiveness. These included using a GPT-4 model trained specifically on sexual health content, a “report AI” button for flagging problematic outputs, and an “ask an agent” feature to allow users to escalate complex or sensitive questions to trained human health educators. Collectively, these ideas reflect a broader, teenager-centered vision for Roo—not merely as a chatbot, but as an integrated, trustworthy health information platform designed to meet the expectations of digitally savvy teenagers. This emphasis on transparency, accessibility, and user agency echoed broader themes across YAC feedback, highlighting a shared desire for efficient, affirming, and user-friendly sexual health resources.

#### Inclusive and Accurate Language Use

Language emerged as a significant concern, particularly with some responses defaulting to cisgender or heteronormative assumptions. For instance, a phrase such as “Does it hurt for girls to have sex for the first time?” was flagged as exclusionary by participants, who noted that such wording could alienate gender-diverse users. Teenagers highlighted the importance of using gender-neutral language to ensure that Roo feels inclusive and affirming for all users, regardless of gender identity.

*I think it’s important it be able to answer questions even if they’re phrased in a gender essentialist way–a young cis girl is probably going to ask something like that rather than ‘ does it hurt to have sex for the first time if you have a vagina.’*

[P6, 4 likes]

As P6 noted, this included rephrasing responses to be both inclusive and accurate, such as changing the example to “Is it painful for people with vaginas to have sex for the first time?” Incorporating such thoughtful language choices was seen as a key step toward making the tool more accessible and welcoming for LGBTQ+ teenagers, while still providing accessible answers for cisgender heterosexual teenagers.

### RQ3: Transparency, Trust, and AI Literacy

#### Transparency and Limitations of Roo’s AI Capabilities

Participants expressed mixed perceptions of Roo’s trustworthiness as an AI-driven sexual health tool. While many teenagers appreciated Roo’s friendly, nonjudgmental tone—describing it as more approachable than other online sources—concerns emerged about its perceived lack of adaptability and clarity about its underlying technology. Some participants found responses too generic or static, raising doubts about Roo’s ability to handle complex or highly individualized questions. Others were unsure whether they were interacting with an AI, a scripted FAQ, or a live human, which undermined their confidence in the chatbot. As 1 participant explained:

*AI learning models are still young and may not always have what you’re looking for, so I think that a live agent would be a good addition for more complex questions. They would be trained to provide accurate factual information about more complex questions that Roo could not*.

[P9]

Teenagers emphasized the need for Roo to be more explicit about its AI nature and its limitations, advocating for clear disclosures, transparent sourcing, and pathways to escalate questions beyond the chatbot when needed. The ambiguity surrounding Roo’s status, paired with inconsistent depth in responses, left some users hesitant to trust the platform fully.

#### AI Literacy and Critical Perspectives on AI Use

Beyond their critiques of Roo specifically, participants demonstrated a striking degree of AI literacy and sophistication in evaluating the role of conversational AI in sexual health education. Teenagers engaged in nuanced discussions comparing Roo to general-purpose large language models (LLMs) such as ChatGPT. Some participants expressed enthusiasm about ChatGPT’s conversational style, with 1 participant remarking, “Yeah I think almost everyone has a positive attitude to ChatGPT. I like it so much. It’s been really helpful” (P10, 2 likes). Others, however, voiced caution about LLMs’ well-documented tendency to produce plausible-sounding but inaccurate information. As 1 participant warned, “It does literally just lie sometimes… if the AI gives out misleading information, it’s going to have a lot of bad effects on the people who use the service” (P8, 6 likes). Rather than passively consuming information, participants positioned themselves as critical evaluators of AI outputs, demonstrating awareness of risks and suggesting safeguards. Several teenagers even articulated concrete design pathways for improving Roo, imagining how a model fine-tuned on credible sources could balance depth with accuracy.

*If trained specifically on trusted sources… it could be, while not perfect, essentially very in-depth GPT—but rather than general questions, it could be way more in-depth on sexual health.*

[P9]

These discussions revealed a nuanced tension: while teenagers were receptive to the idea of more dynamic, conversational AI, they also demanded safeguards such as expert oversight, transparent sourcing, and clear escalation pathways to human support. Their critical perspectives suggest that LGBTQ+ teenagers are not only capable of evaluating emerging technologies but are poised to play a vital role in shaping safer, more accountable AI-driven health resources.

These critiques not only highlighted teenagers’ ability to interrogate the promises and risks of AI but also offered a roadmap for actionable improvements. In particular, their emphasis on the need for clear disclosures, sourcing, and escalation pathways informed Roo’s subsequent revisions around transparency and live educator integration. Teenagers’ calls for AI trained on trusted content also underscored the importance of expanding Roo’s database and linking responses to vetted resources, changes that Planned Parenthood prioritized in the next iteration of the platform.

### Implemented Changes

In response to YAC feedback, PPFA implemented targeted modifications to Roo between May 2023 and October 2024. Changes fell into 3 primary categories: content expansion, language and tone revision, and transparency and interface improvements.

#### Content Expansion

For transgender and gender-affirming care, PPFA added 15 new responses covering testosterone therapy, chest binding, and navigating health care as a transgender person. These included practical guidance (eg, “Taking testosterone can make your period lighter, less frequent, or make them totally go away”) and access information (eg, “Contact your nearest Planned Parenthood health center to see if they offer gender-affirming hormone therapy”).

For queer-specific sexual practices, new content addressed anal health and safety, clitoral anatomy and function, and sexual practices such as scissoring, providing both pleasure-focused information and safety guidance. Additional entries covered topics that teenagers flagged as absent or insufficient, including sex toy safety, miscarriage, and sexual assault resources.

To address concerns about vague or overly general responses, PPFA incorporated more concrete statistics and contextualized examples throughout Roo’s responses. This shift responded directly to participants’ calls for information that normalizes diverse sexual health experiences while providing actionable, specific guidance rather than platitudes such as “everybody is different.”

#### Language and Tone Revisions

PPFA systematically revised Roo’s language to eliminate gendered assumptions and heteronormative framing while emphasizing affirmation and stigma reduction. Gender-essentialist prompts were rewritten to use gender-neutral phrasing (eg, changing “Does it hurt for girls to have sex for the first time?” to “Is it painful for people with vaginas to have sex for the first time?"). This approach ensured that responses remained accessible to cisgender users while affirming gender-diverse teenagers.

STI-related content was updated to explicitly include diverse sexual practices and partnership types, moving beyond heteronormative assumptions. Tone modifications shifted from clinical risk language to affirming, nonjudgmental statements. For example, anal sex information was reframed to state “Anal sex is healthy when done safely. Using lube, going slow, and avoiding anal sex when you have hemorrhoids can help,” positioning pleasure and safety as compatible rather than opposing concerns. Similarly, STI testing was normalized as routine self-care: “Getting tested is often as easy as peeing in a cup at a health center.”

These revisions addressed participants’ concerns that even well-intentioned content could inadvertently reinforce stigma through framing that emphasized risk, pathology, or normative developmental timelines without adequate context.

#### Transparency and Interface Improvements

To address concerns about Roo’s ambiguous identity and perceived limitations, PPFA implemented several transparency-focused changes. Roo’s introductory text was updated to more clearly identify it as an AI-powered tool with specific capabilities and constraints, directly responding to participants’ confusion about whether they were interacting with a live person, a scripted FAQ, or an AI system.

Recognizing teenagers’ desire for human escalation pathways, PPFA added alternative prompts (eg, “Can I talk to a real person?” and “I want to chat to a human”) to facilitate seamless connection to live health educators through Planned Parenthood’s chat services. While this live chat feature has always been part of Roo’s broader infrastructure, it was temporarily removed from the version used during the Northwestern University study due to research constraints. Nevertheless, participants’ strong interest in live support, evidenced by proposals for “ask an agent” features, reaffirms its value as a critical complement to AI-driven answers for handling complex or sensitive questions.

Further efforts were made to increase Roo’s credibility and transparency through the inclusion of “learn more” links within responses, guiding users to vetted Planned Parenthood materials or trustworthy external sites. This addressed teenagers’ concerns about insufficient sourcing and their desire to verify information or explore topics in greater depth.

While structural changes such as adding a full category-based dropdown system, integrating dark mode, or upgrading to more advanced backend AI models (as suggested by P9) remain under consideration for future development phases, PPFA prioritized user experience improvements that could be implemented rapidly. Conversational pathways were streamlined to reduce the number of steps needed to access frequently asked questions, and the language in suggested prompts was reviewed to avoid heteronormative assumptions.

These enhancements reflect Roo’s continued evolution into a transparent and user-centered tool, one that better communicates its capabilities and limitations while expanding teenagers’ access to reliable, affirming health information. Table 2 summarizes key areas of participant feedback alongside corresponding updates made to the platform, illustrating how LGBTQ+ teenager perspectives directly informed and improved this AI-driven health tool.

**Table 2.** Summary of YAC^a^ feedback and corresponding updates to Roo. The table maps participant-identified limitations across 7 domains (content depth, transgender inclusion, stigma reduction, chatbot functionality, language inclusivity, AI^b^ transparency, and AI literacy) to specific design and content changes implemented by PPFA^c^.

Gap identified	Participant feedback	Implemented changes
Content depth and specificity	Need for more nuanced content on medium-depth questions (eg, sexual assault, sex toys, miscarriages, and queer-specific practices).	Added new entries on these topics to improve coverage of LGBTQ+^d^ experiences.
Gender-affirming care and transgender inclusion	Lack of content on hormone therapy, binding, and transgender health care alienates transgender users.	Introduced content on testosterone, binding, and accessing affirming care.
Reducing stigma in health information	Content felt pathologizing or vague; need for nonjudgmental framing and clear statistics.	Revised tone to reduce stigma, included more examples or statistics to contextualize experiences.
Chatbot functionality	Chat felt like a complicated FAQ^e^; suggested dropdowns, dark mode, GPT-4 backend, and escalation to humans.	Streamlined conversational flow; future consideration for dropdown interface and backend upgrades.
Inclusivity and language use	Gendered or heteronormative language alienated users. Requested gender-neutral rephrasings.	Reviewed or revised language in prompts and answers to be gender-neutral and inclusive.
Transparency of AI identity	Confusion about whether Roo was AI or human reduced trust.	Updated introduction text to clarify Roo’s AI identity and limitations.
AI literacy and oversight	Teenagers raised concerns about hallucinations and lack of sourcing; requested live agent escalation and better links.	Emphasized pathways to live health educators and added "learn more” links to vetted sources.

a YAC: Youth Advisory Council.

b AI: artificial intelligence.

c PPFA: Planned Parenthood Federation of America.

d LGBTQ+: lesbian, gay, bisexual, transgender, and queer.

e FAQ: Frequently Asked Questions.

These updates illustrate how teenager-led feedback can shape not just what information is delivered, but how it is structured, presented, and framed. Importantly, many of the changes implemented (eg, improvements to inclusive language, clearer sourcing, expanded topic coverage, etc) speak to both informational and relational aspects of trust-building in AI-based health communication.

## Discussion

### Principal Results

This study demonstrates how participatory design with LGBTQ+ teenagers can meaningfully shape the development of AI-powered sexual health tools. By partnering with PPFA to gather structured feedback on Roo, an AI-enabled chatbot, we engaged a YAC of LGBTQ+ teenagers in identifying gaps, proposing redesigns, and surfacing concerns about trust, inclusivity, and usability. Participants highlighted several key areas for improvement: the need for more in-depth and affirming content (particularly around transgender health, queer-specific sexual practices, and stigma reduction), more flexible and intuitive interface design, and greater transparency about Roo’s limitations as an AI-driven system.

Teenagers also offered critical insight into the epistemic and ethical challenges posed by rule-based AI systems in health contexts, comparing Roo’s limitations to the conversational fluidity and risks of LLMs such as ChatGPT. Rather than rejecting AI altogether, participants articulated a desire for hybrid systems that combined the responsiveness of AI with safeguards such as vetted content, human escalation pathways, and transparent sourcing. Their contributions not only informed direct improvements to Roo’s content, interface, and tone but also pointed toward broader design principles for teenager-centered, accountable AI in health education.

### Reimagining Trust and the Role of AI in Sexual Health Education

Teenagers’ critiques of Roo centered on its perceived limitations as a rule-based system—an AI approach that relies on preprogrammed responses to anticipated queries rather than dynamically generating answers based on context. While many appreciated Roo’s nonjudgmental tone, they described the platform as rigid, generic, and limited in depth, particularly when responding to nuanced or medium-complexity questions. Participants noted that while rule-based systems can offer predictability and safety, they often struggle to meet users’ evolving or individualized information needs, especially in sensitive areas such as LGBTQ+ sexual health.

Several teenagers compared Roo to more dynamic tools such as ChatGPT, noting that while Roo felt safe and reliable, it sometimes lacked the adaptability or richness of a conversational AI. These critiques mirror broader limitations of digital health resources, which often lack the contextual responsiveness needed to address users’ diverse needs, especially in areas such as mental health and identity. Research has shown that many digital tools fail to provide accessible, personalized, or comprehensible information, limiting their effectiveness in real-world contexts [45]. Although static resources offer advantages in terms of accuracy and control, they often struggle to serve teenagers navigating sensitive, intersectional, or highly personalized sexual health concerns [46,47].

Yet, it is important to balance these concerns with evidence demonstrating AI’s potential promise in health care. Systematic reviews show that conversational agents can effectively deliver health interventions when designed appropriately, with research indicating that chatbots can improve patient engagement, provide timely information access, and demonstrate acceptable accuracy [48]. Studies of user acceptance show that when people believe AI technology will be useful, this can serve as a strong predictor of adoption among LGBTQ+ individuals [49]. In contrast, dynamic and interactive tools (eg, chatbots or AI-based conversational agents) offer real-time responsiveness and perceived personalization, which may foster deeper engagement and affirmation, particularly for LGBTQ+ teenagers seeking information not readily reflected in mainstream health education [50].

Our findings extend this literature by demonstrating how trust is co-constructed through participatory design processes that enable users to contest and refine system outputs. While participants raised concerns about Roo’s limitations, research demonstrates that conversational agents can be highly effective in health care when designed with transparency and accountability in mind [48,51]. The framework by Shneiderman [52] for trustworthy AI emphasizes the importance of human control and oversight, principles echoed in teenagers’ proposals for “report AI” buttons and live agent escalation. Our findings extend this framework by demonstrating how trust emerges not from system perfection but from honest acknowledgment of constraints coupled with meaningful avenues for user input and recourse.

These expectations for responsiveness were closely tied to broader notions of trust. Existing research suggests that teenagers are more likely to trust AI health tools when systems clearly communicate their limitations, provide opportunities for human escalation, and demonstrate credibility through transparent sourcing [53]. Liao and Sundar [54] argue that designing for responsible trust in AI systems requires clear communication strategies that help users understand system capabilities and limitations. Trust was not predicated simply on whether a system used AI, but on how well it acknowledged uncertainty, supported user agency, and created channels for recourse when information felt insufficient or inaccurate. In this sense, trust and accountability were deeply intertwined: participants wanted tools they could rely on, not because they were infallible, but because they made space for users to push back, seek clarification, and surface unmet needs.

At the same time, participants demonstrated striking AI literacy. Teenagers critically discussed the strengths and risks of integrating LLMs such as ChatGPT into health tools, raising concerns about hallucinated facts, citation gaps, and the overconfidence of generative outputs. While some valued the conversational range offered by ChatGPT, others emphasized the potential harm of inaccurate information in high-stakes health contexts. Rather than rejecting AI altogether, participants proposed pragmatic solutions, such as fine-tuning models on verified health content, linking outputs to reputable sources, and integrating moderation or “report” features that would allow users to flag problematic responses and request additional review. These reflections signal that LGBTQ+ teenagers are not only capable of identifying epistemic risks but are actively imagining systems of accountability. Their feedback points toward a broader vision of participatory AI governance, one in which teenagers are positioned not as passive users, but as costewards of tools designed for their well-being.

### Centering Teenage Voices to Advance Equity in AI-Enabled Health Tools

Roo is one of the few publicly available AI-powered chatbots designed to provide on-demand sexual health information to teenagers. Unlike static websites, Roo offers scalable, interactive engagement across a broad spectrum of questions, positioning it as a unique resource within an otherwise sparse landscape of LGBTQ+ inclusive digital health tools. Roo was developed in part to address the information marginalization faced by LGBTQ+ teenagers whose needs are often excluded from mainstream sexual health education. Through this project, Roo incorporated content specifically tailored to queer and transgender teenagers, including information on gender-affirming care, STI stigma, and mental health in sexual contexts. Yet despite these intentions, participants identified ongoing limitations in Roo’s ability to represent and respond to the full scope of their lived experiences. Feedback highlighted not just content gaps, but structural constraints, such as rigid dialog flows and static language, that limited Roo’s responsiveness, particularly for users with intersectional or stigmatized identities. These observations reinforced a critical lesson: content inclusivity is necessary, but not sufficient, for digital equity. The form and functionality of the system must also reflect the realities and needs of those it intends to serve.

To that end, Roo’s participatory development process offers an important model for advancing equity in AI-based health interventions. Our engagement with the YAC gestures toward what Constanza-Chock [55] terms “design justice,” in its effort to foreground the experiences of those most affected by the technology. LGBTQ+ teenagers were treated as co-designers whose insights materially shaped both the chatbot’s interface and content. Their feedback drove refinements to language, layout, and tone, ensuring that Roo felt accessible, affirming, and useful to those it was built for. Participants’ sophisticated critiques align with the observation by Yang et al [56] that human-AI interaction presents unique design challenges that require iterative, user-centered approaches. By positioning teenagers as epistemic contributors, we demonstrate how participatory methods can address the guidelines for human-AI interactions proposed by Amershi et al [57], particularly around transparency, user control, and system adaptability. These design contributions align with research showing that participatory methods, particularly when embedded early and iteratively, can produce tools that are not only more usable but also more trusted and ethically aligned [55]. Beyond improving Roo itself, the process also demonstrated how co-design with marginalized teenagers can serve as a form of epistemic justice: making space for community knowledge to shape what counts as valid, visible, and valuable in digital systems.

Importantly, this process also cultivated a form of applied AI literacy among participants. Teenagers evaluated its limitations, questioned its logic, and proposed sophisticated alternatives, such as integrating GPT-4 with guardrails or flagging outputs for review. Engaging teenagers in the design process encouraged them to question the intelligence and trustworthiness of AI, equipping them with the skills to navigate the complexities of digital health technologies. This aligns with the growing recognition that AI literacy is essential for empowering marginalized populations to not only use AI tools effectively but also challenge their limitations [58]. By blending participatory design with transparent and inclusive AI practices, this study highlights a replicable model for fostering critical engagement with AI—one that enables LGBTQ+ teenagers to transition from passive users to informed, empowered stakeholders.

### Privacy and Confidentiality Considerations

Interestingly, while participants raised concerns about content accuracy and trustworthiness, explicit concerns about data privacy were not prominent in their feedback. This finding contrasts with research suggesting that LGBTQ+ teenagers often prioritize anonymity and privacy in online health-seeking [59]. The relative absence of privacy concerns may reflect Roo’s design as a web-based tool requiring no account creation or personal information, or it may suggest that content quality and inclusivity were more salient concerns than data practices. Roo’s design incorporates privacy-protective features to encourage honest engagement: no account creation, login, or personal information is required, and users access the platform anonymously via web browser. However, this study did not directly assess whether teenagers experienced self-censorship despite these protections. Future research should examine whether privacy concerns shape the types of questions teenagers feel comfortable asking AI health tools.

However, this should not be interpreted as evidence that privacy is unimportant to LGBTQ+ teenagers accessing sexual health information. The broader literature on digital health emphasizes that privacy concerns intersect with identity safety and institutional trust, particularly for populations experiencing health inequities [60]. For LGBTQ+ teenagers who may face family rejection or school-based discrimination, privacy in health-seeking is not merely a preference but a safety imperative. Future research should explicitly probe privacy expectations and concerns among LGBTQ+ teenagers using AI health tools, particularly as these systems become more personalized and data-intensive.

### Implications for Human-Centered AI, Health Informatics, and Public Health

This work contributes to human-computer interaction scholarship by demonstrating how participatory design with marginalized teenagers can surface unique requirements for AI systems that serve vulnerable populations. While guidelines for human-AI interaction emphasize transparency and user control, our findings reveal additional considerations specific to LGBTQ+ teenagers: the need for affirming language, intersectional content, and explicit acknowledgment of AI limitations [57]. These insights extend existing human-computer interaction frameworks and suggest that design guidelines must be iteratively refined through engagement with diverse user communities.

From a health informatics perspective, our findings corroborate observations that health chatbots require improvements in content quality and engagement depth, while extending this to show that effectiveness depends on attending to relational and cultural dimensions of care [61]. The emphasis participants placed on inclusive language, transgender-specific content, and stigma-reducing framing highlights that health information systems must address both informational accuracy and the social contexts in which health information is sought and used.

For public health practice, this study underscores AI’s potential to address health disparities when development processes are actively centered around marginalized communities. However, realizing this potential requires sustained participatory engagement rather than 1-time consultation, as demonstrated by Madaio et al [62]. Co-designing checklists and interventions with affected communities helps organizations understand and address bias in AI systems while building trust directly reflected in our partnership model.

### Lessons Learned From Academic and Nonprofit Partnership

This project exemplifies the value of cross-organizational collaboration in advancing equitable digital health tools, while also surfacing important lessons for future partnerships.

#### Complementary Organizational Capacities Accelerate Impact

Universities and nonprofits bring distinct but synergistic strengths. Academic institutions offer methodological expertise and research infrastructure; nonprofits bring public health infrastructure and capacity for rapid implementation. This partnership demonstrated how combining these capacities collapsed the research-to-practice timeline, with teenage feedback translated into platform updates within months rather than years.

#### Early Alignment on Values and Decision-Making Prevents Conflict

Establishing clear agreements about decision-making authority was essential. Northwestern University maintained control over research design and analysis; PPFA retained authority over content implementation. This division preserved both scientific integrity and organizational autonomy but required extensive upfront communication and shared commitment to health equity values. Organizations considering similar partnerships should assess both structural agreements and value compatibility early.

#### Partnership Infrastructure Requires Dedicated Resources and Sustained Engagement

While this model offers significant advantages, it demands substantial coordination that is often unfunded or underestimated. Faculty time for relationship-building, legal negotiations, and ongoing communication represents real costs, as does nonprofit capacity for research collaboration that may compete with direct service priorities.

### Implications for Practice and Future Directions

This study highlights both the promise and complexity of developing AI-enabled health education tools for LGBTQ+ teenagers. Roo demonstrates how chatbots can expand access to affirming, immediate, and nonjudgmental information, particularly for teenagers navigating sensitive, stigmatized, or nuanced questions. For sexual health education in particular, questions often defy simple rule-based answers and demand context, empathy, and nuance. Our findings suggest that participatory design should not end once a tool is launched; instead, teenagers must be meaningfully engaged in ongoing feedback, evaluation, and refinement cycles. Designing for accountability means enabling mechanisms for teenagers to contest, correct, or expand system responses, and treating these interventions not as critiques to be managed, but as vital contributions to more inclusive, responsive AI systems.

This study also reinforces the value of embedding participatory design as a continuous, rather than episodic, element of tool development. LGBTQ+ teenagers brought critical perspectives that shaped Roo’s language, tone, content depth, and usability, insights that could not have emerged without their sustained involvement. Importantly, the co-design process also cultivated AI literacy, empowering teenagers to assess the risks and capabilities of emerging technologies. As generative AI tools proliferate in health and education settings, there is an urgent need for implementation models that support participatory governance, foster critical engagement, and include marginalized teenagers not only as users but as epistemic contributors. Future efforts might explore how such participatory frameworks can be institutionalized across digital health product life cycles, from ideation to deployment to postlaunch evaluation.

Scalability will also require stronger cross-sector partnerships. This project benefited from the combined expertise of a public health nonprofit and a university-based research team, enabling agile iteration while preserving scientific rigor. Finally, more research is needed to evaluate the long-term impact of AI-based sexual health tools on teenage health outcomes, particularly among queer and transgender teenagers. Future studies should explore not only what works, but for whom, under what conditions, and with what ethical guardrails in place.

### Limitations

While our YAC included participants across gender identities and races or ethnicities, the sample was limited. Of 15 participants, 9 (60%) identified as White, suggesting recruitment methods may have inadvertently excluded some racial and ethnic minority teenagers whose perspectives may differ regarding institutional trust and health care bias. The sample size was relatively small, and participants self-selected into this study, introducing potential bias toward those already motivated to engage with sexual health tools. While we examined how gender identity shaped experiences with Roo, we did not systematically analyze how histories of gender-based discrimination—including transphobia and transmisogyny—influenced teenagers’ trust in AI health tools. Future research should intentionally oversample LGBTQ+ teenagers of color using culturally responsive methods and explicitly examine how systemic marginalization informs interactions with AI platforms.

The customized instance of Roo used during this study differed from the publicly available version—most notably by removing the live chat feature due to research-related monitoring constraints. This may have influenced perceptions of trust, usability, and comprehensiveness. Additionally, this study did not capture or analyze individual conversation logs between participants and Roo, instead relying on participants’ reflections posted in Discord discussions. While this preserves privacy and reduces data burden, future studies might benefit from analyzing actual conversation data (with consent) to identify specific interaction patterns or content gaps not fully captured through retrospective self-report.

Although our YAC discussions did not capture which specific sources teenagers consider trustworthy, prior work suggests that teenagers often place greater confidence in well-established health organizations, including the Centers for Disease Control and Prevention and the World Health Organization, when information is perceived as transparent, relevant, and easy to understand [63]. At the same time, marginalized teenagers may be more likely to trust organizations with reputations for inclusive and affirming care, such as Planned Parenthood, which align more closely with their lived experiences and needs [64]. Future research should directly examine which health information sources LGBTQ+ teenagers consider credible, to better inform how chatbots such as Roo can direct users to external resources.

Finally, while our academic and nonprofit partnership enabled rapid feedback translation and impact, it also surfaced challenges around legal, operational, and infrastructural coordination. Negotiating data use, content ownership, and design authority required extensive collaboration, and the scalability of this model may depend on the capacity and alignment of future partnering institutions. Nonetheless, this case offers a valuable proof of concept for how cross-sector partnerships can meaningfully center community voices in AI health tool development.

### Conclusions

This project illustrates the importance of designing AI-enabled health tools not just for marginalized teenagers, but with them—grounding development in participatory design, cross-sector collaboration, and iterative accountability. Roo’s evolution through feedback from LGBTQ+ teenagers highlights the critical need to center those most affected by information gaps in shaping both the content and infrastructure of digital health tools. More than just expanding access to stigma-free sexual health information, Roo’s development process surfaced deeper questions about how AI systems earn trust, respond to critique, and evolve alongside community needs. By treating teenagers as epistemic contributors, this work offers a model for how AI can be governed more ethically, transparently, and equitably. The collaboration between Northwestern University and Planned Parenthood demonstrates how academic and public partnerships can meaningfully integrate community voices into scalable interventions that are not only technically sound but socially accountable.

## Abbreviations

AI: artificial intelligence
G2G: Guy2Guy
LGBTQ+: lesbian, gay, bisexual, transgender, and queer
LLM: large language model
PPFA: Planned Parenthood Federation of America
REDCap: Research Electronic Data Capture
RQA: rapid qualitative analysis
SHER: Sharing Health Education Resources
STI: sexually transmitted infection
YAC: Youth Advisory Council
