# Correction: Enhancing LGBTQ+ Inclusivity in an AI-Powered Sexual Health Chatbot: User-Centered Design Approach Through a Nonprofit and Academic Partnership

**DOI:** 10.2196/96639

**Published:** 2026-05-04

**Authors:** William Wibowo Liem, Elizabeth Casline, Julianna Lorenzo, Jacob D Gordon, Andres Alvarado Avila, Attia Taylor, Nicole Levitz, Michael C O'Keefe, Karen Shum, Kathryn Macapagal

**Affiliations:** 1 Impact Institute, Northwestern University, Chicago, IL, United States; 2 College of Nursing, University of Cincinnati, Cincinnati, OH, United States; 3 Planned Parenthood Federation of America, New York, NY, United States; 4 Department of Medical Social Sciences, Impact Institute, Northwestern University, 625 North Michigan Avenue, Floor 14, Chicago, IL, United States, 1 312 503 3605

In “Enhancing LGBTQ+ Inclusivity in an AI-Powered Sexual Health Chatbot: User-Centered Design Approach Through a Nonprofit and Academic Partnership” [1], the authors made one addition.

Author KS was inadvertently omitted from the authorship list. They have been added as the ninth author as follows:

Karen Shum, BA^3^

ORCID: 0009-0004-0094-4818

3. Planned Parenthood Federation of America, New York City, NY, United States

In the Authors’ Contributions section, the following was added:

KS reviewed the manuscript and provided approval of the final work.

The correction will appear in the online version of the paper on the JMIR Publications website, together with the publication of this correction notice. Because this was made after submission to PubMed, PubMed Central, and other full-text repositories, the corrected article has also been resubmitted to those repositories.
